# Supplementary material for: Experiences of public-private contracting for caesarean delivery in rural district public hospitals: A qualitative interview study
Source: PLOS Glob Public Health. 2023 May 8;3(5):e0001335. doi: 10.1371/journal.pgph.0001335 (PMC10166521; doi:10.1371/journal.pgph.0001335)
Supplement: S1 Table — (DOCX) [file pgph.0001335.s001.docx]

S1 Table: Interview guide questions organised according to the framework[1] of drivers, challenges and required action for obstetric care in preparing for national health insurance

| Area | Sub area | Interview guide questions |
| --- | --- | --- |
| Women and community factors | 1. Prevalence of medical conditions for which CS would be indicated | Can women themselves decide to have a caesarean delivery on request?  What is your caesarean rate in this hospital; and do you think it is too high or too low? What drives this rate? |
| Health professional factors | Training | Do doctors or nurses in your hospital have the skills to perform assisted vaginal deliveries e.g. Forceps and Ventouse?  What is your primary need to improve the provision of maternity care in this hospital? |
|  | Remuneration model | When did you first enter into a contract with government?  What kind of contract do you have currently? (probe sessions, fee for service etc.)  How do you feel about the contract you currently have (probe: fairness of remuneration) |
|  | Medico-legal concerns | Do you think medico-legal concerns influence decision making around caesarean delivery?  How are you indemnified against medico-legal claims when you work in a public health facility? Probe Medical Protection Society or government insurers? |
| Organizational and system factors | Models of care | Can you tell us about how maternity care works in your hospital e.g. do midwives or doctors do vaginal deliveries, when do doctors get involved in labour ward care?  Who makes the decision to perform a caesarean delivery?  What are the advantages for you of entering into a contract with a government hospital?  What are the advantages of bringing private providers into government hospitals?  What are the challenges of bringing private providers into government hospitals? How can these be overcome?  Are there any circumstances when there might be benefit in sending some of your patients to a private facility (e.g. lack of beds or theatre space, or lack of skill)? |
|  | Human resource availability | Do you feel you have adequate human resources for performing caesarean deliveries in this hospital (probe for surgical and anaesthetic capacity)?  What level of provider performs the surgery and who provides anaesthesia?  Does your hospital have scheduled and time tabled provision of service from private providers. If yes, probe what prompted this arrangement, what needs does it address?  What services are private GPs contracted to provide?  Tell me about the kinds of cases that you are called to manage (probe: complicated NVDs, complicated caesarean deliveries, elective caesarean deliveries etc) |

Reference:

1. Solanki GC, Cornell JE, Daviaud E, Fawcus S. Caesarean section rates in South Africa: A case study of the health systems challenges for the proposed National Health Insurance. SAMJ. 2020;110(8).
