# Supplementary material for: Experiences of public-private contracting for caesarean delivery in rural district public hospitals: A qualitative interview study
Source: PLOS Glob Public Health. 2023 May 8;3(5):e0001335. doi: 10.1371/journal.pgph.0001335 (PMC10166521; doi:10.1371/journal.pgph.0001335)
Supplement: S2 Table — (DOCX) [file pgph.0001335.s002.docx]

S2 Table: Example of the process of data analysis to develop codes, sub-categories, categories and themes

| Theme | **Retaining skilled medical staff who can perform caesarean deliveries in rural district hospitals** | | |
| --- | --- | --- | --- |
| Category | Training | Staff turnover | Private sector stability |
| Sub-category | Junior medical staff | Human resource recruitment delays | GP contracting |
| Codes | Private sector resources  ‘’*the only senior experts or doctors is your GPs’’* | caesarean delivery skill loss  ‘’*Especially if you lose your staff that can do caesars’’* | Relationship building  ‘’*you should try and establish a very good working relationship with the skills that's available in a town’’* |
