# Supplementary material for: Experiences of public-private contracting for caesarean delivery in rural district public hospitals: A qualitative interview study
Source: PLOS Glob Public Health. 2023 May 8;3(5):e0001335. doi: 10.1371/journal.pgph.0001335 (PMC10166521; doi:10.1371/journal.pgph.0001335)
Supplement: Attachment — Submitted filename: Response to reviewers Plos GPH.docx [file pgph.0001335.s004.docx]

Dr Patrick A. Palmieri

Academic Editor

Plos Global Public Health

Dear Dr Palmieri

**Response to reviewer comments on PGPH-D-22-01557**

Thank you for the reviewer comments on our manuscript. We found these comments helpful in strengthening the paper and have responded point by point to the comments below.

Editor comments:

There are multiple observations to address limitations in reporting the study and to improve the clarity of presentation. When the authors undertake the revisions, the SRQR (Standards for reporting qualitative research) checklist should be applied to the manuscript to make sure all the recommended reporting elements have been properly addressed ([https://www.equator-network.org/reporting-guidelines/srqr/](https://protect-za.mimecast.com/s/F_oXCJZKVAIp55QPHGG4D2)). The completed SRQR checklist needs to be returned with the revised manuscript with the page and line or paragraph numbers indicated for each reporting element. Please make sure to review the supporting document for the checklist to make sure ALL the criteria for each element are properly addressed.
*Response: Thank you, we have uploaded the SRQR checklist with the revised manuscript*

Reviewer #1:

To the best of my knowledge, the manuscript meets PLOS Global Public Health's publication criteria.
All data pertaining to the manuscript has been provided except for transcripts which the authors retained for participant privacy.
The manuscript is well written, however, the authors should format paragraphs by justifying so that the text is distributed evenly within the margins.
Under the methods section, I advise authors to format subtitles by making them bold.
To have a better read, more punctuation should be done by the authors, especially the use of commas.

*Response: Thank you we have addressed these formatting suggestions as per PLOS guidelines.*

Reviewer #2:

This article explores the challenge faced by many LMICs in the pursuit of health care service provision for the population. The authors explore the experiences and perceptions of providers in private and public health care facilities to public-private contracting for caesarean delivery in rural district public hospitals in the Western Cape Province of South Africa.
There are several recommended areas of improvement
It would have been useful to have line numbers and page numbers in the manuscript

*Response: we have added page numbers to the manuscript*

Introduction
• Please include statistics on the uninsured population that use the private sector and the insured population that use the public sector services

*Response: This has been added on page 3.*

• Is there any available financial data on the costs of public private contracting in South Africa

*Response: To our knowledge there are no other public-private contracting initiatives in South Africa that involve contracting in private providers to provide services in public health facilities. Other public-private partnerships in the health sector in South Africa are all infrastructure projects to build hospitals in five provinces and one state vaccine institute. This has been included on page 4.*

• The authors indicate there has been a decline in maternal mortality. It would be useful to indicate during what time frame this has occurred and the earlier material mortality rates.

*Response: This has been added on page 3.*

• It is unclear if the high caesarean delivery rate in the private sector is considered a pattern of inappropriate care (this paragraph last two sentences)

*Response: We have amended this sentence to make it clear that the high caesarean delivery rate is considered inappropriate care (page 3)*

• Can the different models of PPEs documented in LMICs be described in greater detail, perhaps with specific examples from other countries.

*Response: we have included examples of PPEs for obstetric care in India. We have found no published research on PPEs for obstetric care in Africa. This has been included on page 4.*

Under Methodology
• What is the population of this province?

*Response: This has been added on page 4.*

• How many deliveries occur in the private facilities and how many in the public facilities?

*Response: we have added the number of deliveries in public health facilities in the Western Cape province on page 5. Data is not available for deliveries occurring in private health facilities as this data is owned by the private hospital groups.*

• How many caesarean deliveries take place in the private facilities and how many in the public facilities in the province?

*Response: We have added the caesarean delivery rate in public health facilities in the Western cape on page 5. There is no available data on caesarean delivery rates for private hospitals in the Western Cape. The national caesarean delivery rate has been cited in the introduction on page 3.*

• How many district hospitals and regional hospitals were in this province? How many regional hospitals are in this province?

*Response: This has been added on page 4.*

• Were these five district hospitals selected because they were the only ones that had existing contracting arrangements?

*Response: Yes, these were the only district hospitals at the time of the study that had existing contracting arrangements with private GPs. This has been clarified on page 5.*

• Were the hospitals labelled A, B, C, D, E, and F? Which ones were the district hospitals, and which one is the Regional Hospital?

*Response: Yes this is the descriptor used to provide anonymity for the hospitals. A-E are the district hospitals and F is the regional hospital. This has been clarified on page 7.*

• What language was used in the interview? Was there any translation of the transcripts required?

*Response: Interviews were all conducted in English and no translation was required. This has been added on page 6.*

• Was any prior literature used to develop the interview questions?

*Response: In developing the interview guides we drew on a framework previously developed by members of our research team which outlines drivers, challenges and required action for obstetric care in preparing for NHI. This has been clarified on page 5&6.*

• What types of questions were included in the interview schedule?

*Response: We have included examples of the types of questions included in the interview guides on page 6.*

• What were the selection criteria for participants?

*Response: we have described the selection process for interviews on page 6.*

• What were the roles of the 3 district managers mentioned under data collection? In the results section quotes are attributed to District Director, District Human Resource Manager, District Finance Manager (hospital affiliation not provided) , Medical Manager, Manager Rural Health Services and Hospital Manager. Please clarify.

*Response: we have now clarified this in table 1. There were 4 interviews with district management. They are all based within district offices and not at a specific hospital. We have included these details on page 6.*

• Where was the interview conducted at the facilities? Was it a private room?

*Response: we have added this information on page 6.*

• Interview duration (range of time and average length of time)?

*Response: we have added this on page 6.*

• What measures were taken to ensure confidentiality? Did the interviewees receive an honorarium for their participation?

*Response: we have clarified these two points on page 7.*

• Who transcribed the interviews (a team member or a RA)?

*Response: a professional transcriber transcribed the interviews which were check against audios for accuracy by two members of the research team. This has been added on page 7.*

Results
• A small table which breakdowns the gender, role and facility would be useful for the reader

*Response: we have added a table (table 1) outlining the interviewees per facility, gender and whether a private GP or government employed doctors.*

• Outline in the first paragraph the six thematic areas that would be covered in the results section. Not all are mentioned

*Response: Thank you, we have re written the first paragraph of the results to briefly outline the six thematic areas. Page 7.*

• It is unclear if all of the interviewees shared similar opinions and if both subcategories of doctors are included, eg “Public sector hospital manager and doctors described the justification for entering into contracting agreements with private GPs in their towns” Does doctors refer to both private sector and government employed doctors? Did all of the doctors interviewed justify contracting agreements with private GPs in their towns? There were no quotes from government employed doctors under the theme.

*Response: we have amended the text to more clearly ascribe the quotes to specific categories of interviewees on page 8. We have also added a quote from a government doctor on page 8.*

• Clarify which quotes emerged from personnel at district hospitals or the regional hospitals

*Response: this has now been clarified with the addition of table 1. The district hospitals are hospitals A-E and the regional hospital is hospital F.*

• Please provide numbers alongside some, several to clarify for the reader how many of the interviewees shared this view.

*Response: we have amended text throughout the results section to be more specific regarding participant roles and views.*

• There is an observably higher representation of quotes from private GPs and managers compared to government employed doctors in the results section. Recommendation to include quotes from all categories of interviewees.
o 4 quotes from Government employed doctors – medical officer Hospital D, medical officer Hospital B, Obstetrician from Hospital F (two quotes attributed)
o 14 quotes from Private GPs – 7 from private GP at hospital C, 5 from private GP at hospital B, 1 from private GP at hospital A and one from private GP at hospital D
o 12 quotes from manager level

*Response: with the addition of table 1 we have clarified the distribution of interviewees across hospitals. In one hospital there were no government employed doctors as a private GP practice was contracted to run the hospital. Hence overall we interviewed more private GPs than government employed doctors. For some of the major themes relating to the advantages of contracting with government the views are solely those of the private GPs and regarding financial reasons for PPE the district managers opinions dominate since they are at the level where financial and contracting decisions are made. With this revision we have tried to bring more balance in terms of the views of government employed doctors.*

Discussion
This section would be improved by comparing the results with the perceptions of and models of PPEs utilized in other LMICs, or other provinces in South Africa. This can include the financial impact of utilizing PPEs on government’s health care expenditures, levels of efficiency, public acceptance and health statistics. Please provide statistics and references where needed.

Response: *To our knowledge there are no other public-private contracting initiatives in South Africa that involve contracting in private providers to provide services in public health facilities. Other public-private partnerships in the health sector in South Africa are all infrastructure projects to build hospitals in five provinces and one state vaccine institute. This has been included on page 4. We have included additional relevant literature on contracting out health services to improve the discussion on page 15. There is very little published research on PPEs for maternity care in low and middle income countries.*

In South Africa, the private insured population in rural communities is lower than in cities which limits the earning potential of private practice (statistics and citation).

*Response: we have added data regarding medical insurance coverage to support this statement on page 16.*
