# Supplementary material for: Experiences of public-private contracting for caesarean delivery in rural district public hospitals: A qualitative interview study
Source: PLOS Glob Public Health. 2023 May 8;3(5):e0001335. doi: 10.1371/journal.pgph.0001335 (PMC10166521; doi:10.1371/journal.pgph.0001335)
Supplement: Attachment — Submitted filename: Response to reviewers Plos GPH.docx [file pgph.0001335.s005.docx]

Miquel Vall-llosera Camps
Staff Editor
PLOS Global Public Health

Dear Dr Vall-llosera Camps

**Response to reviewer comments on PGPH-D-22-01557**

Thank you for the second round of reviewer comments on our manuscript. We found these comments helpful in strengthening the paper and have responded point by point to the comments below.

Reviewer #3:

Thank you for the opportunity to review the article "Perspectives of public-private contracting for caesarean delivery in rural district public hospitals: A qualitative interview study" submitted to PLOS Global Public Health. The majority of the feedback for this review is provided in the marked version of the manuscript pdf to assist the authors with the recommended revisions.

*Response: Thank you for the marked up document. We have gone through and made revisions in response to comments. Where we need to explain a revision we have included those comments in this document below.*

The introduction can be improved with editing and minor revisions, possibly some rearranging, as recommended in the marked version of the manuscript.

*Response: thank you we have made the changes as suggested in the marked up document.*

Also, there seems to be some information in the methods section that is more specific to the background than to the methods.

*Response: we have created a sub heading ‘sampling’ and moved some of the information regarding the selection of hospitals there. The description of the district hospitals we have left under ‘setting’ as we feel it fits best there.*

The methods section requires multiple revisions to comply with the reporting elements of the SRQR which was attached as the reporting guideline. For example, the information about trustworthiness is largely not reported in the methods section. More importantly, the data analysis area requires improvement as the process outlined by Graneheim & Lundman is largely not reflected in the current description of the study. Similarly, the SRQR addresses the coding tree, an important feature in the process described by Graneheim & Lundman (see figures 1 and 2).

*Response: we have done major revisions to the methods section to comply with SRQR reporting requirements including the addition of two supplementary tables.*

The discussion section requires more information about the similarities and differences in the findings of this study with other studies, from LMICs as well as HICs.

*Response: Through our extensive literature review we could find no other research exploring PPEs for obstetric care, utilising non specialist clinicians in Africa. This is an important finding which justifies the relevance of this research. We have revised the discussion to emphasise the lack of previous research and implications of these findings and have brought in additional literature.*

The current discussion is mechanical in summarizing the findings with little insightful comparisons from the larger literature. As a notation, the "paucity of literature" evidenced by the authors with the Cochrane review is incorrect due to the inclusion criteria. In this regard, the current study would not be included in the cited Cochrane review.

*Response: Our intention was not to compare our findings to the Cochrane review it was to make the point that there exists very little published research on PPEs involving contracting of medical staff for hospitals in the African setting.*

Finally, the manuscript lacks clarity in some areas due to the wordy, and often long, sentences. For this reason, the manuscript requires a thorough editing to produce clear and concise sentences. Multiple examples are provided in the attached pdf. Please keep in mind the feedback in the attached pdf is quite critical, but in a constructive manner to address the observations, limitations, and recommendations. Once the revisions are completed, the article should be a good contribution to the literature.

*Response: Thank you we have done a through edit throughout to improve the readability.*

**Additional responses to comments in the marked-up file where appropriate:**

Please change the word doctor to physicians throughout the manuscript.

*Response: we have changed the word ‘doctor’ to ‘General Practitioner’ throughout the manuscript when referring to the private GPs. Physician in South Africa is often the term used to describe an internal medicine specialist. General Practitioner is a term that should be understood by a global audience. We use the term medical officer in relation to government employed doctors in the district hospitals since this is their official title.*

Was data from this study already published? Will this qualitative data be published again in another manuscript?

*Response: A paper utilising the quantitative data on obstetric outcomes has been accepted to BMJ Open. We have added a citation for this paper. The qualitative data relating to the specific contracting models will be used in a third paper that specifically presents the contracting models used across these five district hospitals. There is no repetition of interview quotes across the papers which each have a distinct focus.*

A large portion of this information should be provided in the background for the study rather than the setting.

*Response: As suggested we have created a new sub heading called ‘sampling’ where we have moved the description relating to why these five hospitals were sampled. The remaining description of the services and human resources at district hospitals we feel does belong under study setting as it relates specifically to the context in the study hospitals and would not fit well in the introduction which presents the study topic and relevant literature.*
